# Therapeutic Effects of Static Magnetic Field on Wound Healing in Diabetic Rats

**DOI:** 10.1155/2017/6305370

**Published:** 2017-03-26

**Authors:** Jing Zhao, Yong-guo Li, Kai-qin Deng, Peng Yun, Ting Gong

**Affiliations:** ^1^Central Laboratory, Medical School of Yangtze University, Hubei 434003, China; ^2^Department of Endocrinology, Central Hospital of Jingzhou City, Hubei 434001, China; ^3^Department of Endocrinology, The First Affiliated Hospital of Yangtze University, Hubei 434000, China; ^4^Department of Internal Medicine, The First Clinical Medical School of Yangtze University, Hubei 434000, China

## Abstract

*Objective*. To investigate the effects of static magnetic field (SMF) on cutaneous wound healing of Streptozotocin- (STZ-) induced diabetic rats.* Methods*. 20 STZ-induced diabetic rats were randomly divided into two groups (10 in each group): diabetic rats with SMF exposure group which were exposed to SMF by gluing one magnetic disk of 230 mT intensity and diabetic rats with sham SMF exposure group (sham group). 10 normal Wistar rats were used as the control group. One open circular wound with 2 cm diameter in the dorsum was generated on both normal and diabetic rats and then covered with sterile gauzes. Wound healing was evaluated by wound area reduction rate, mean time to wound closure, and wound tensile strength.* Results*. The wound area reduction rate in diabetic rats in comparison with the control group was significantly decreased (*P* < 0.01). Compared with sham magnet group, diabetic rats under 230 mT SMF exposure demonstrated significantly accelerated wound area reduction rate on postoperative days 7, 14, and 21 and decreased gross time to wound closure (*P* < 0.05), as well as dramatically higher wound tissue strength (*P* < 0.05) on 21st day.* Conclusion*. 230 mT SMF promoted the healing of skin wound in diabetic rats and may provide a non-invasive therapeutic tool for impaired wound healing of diabetic patients.

## 1. Introduction

Diabetes mellitus (DM) is a metabolic disorder that is characterized by chronic hyperglycaemia. It is a common and potentially disabling chronic disease. The condition is presently afflicting 382 million people worldwide [1]. This rise in prevalence of DM is likely to bring a concomitant increase in its complications among diabetic patients. One important complication of DM is the diabetic skin ulcer. Impaired wound healing of skin ulcer in diabetic patients is a common, serious and costly global health issue. It is a leading cause of admission, amputation and mortality in diabetic patients [2]. How to accelerate the wound healing in diabetic patients and relieve their suffering has become a great challenge to medical field. Static magnetic field (SMF) as alternative noninvasive method can produce satisfying therapeutic effects on different kinds of tissue defects [3, 4]. Previous study has showed that 220 mT SMF increased the rate of healing by secondary intention in normal rats [5], but few study to date has examined SMF effect on diabetic wound healing. In this study, we examined the effects of an externally applied electromagnetic field, a 230 mT SMF generated by a permanent NeFeB magnet, to investigate the effects of SMF on cutaneous wound healing in Streptozotocin (STZ)-induced diabetic rats.

## 2. Methods

### 2.1. Experimental Diabetes

26 adult male Wistar rats were provided by Animal Center of Wuhan University (Wuhan, China) and housed in a room with Controlled temperature (23 ± 1°C), relative humidity (50–60%), and alternately light-dark cycle (12 h/12 h), with access to standard pellet and clean water. Diabetes mellitus was induced by a subcutaneous injection of 65 mg/kg streptozocin (Sigma Chemicals, St. Louis, MO, USA; freshly dissolved in sterile saline, 0.9%). Confirmation of hyperglycemia was made three days after STZ injection, and only STZ treated rats whose glucose concentration of the tail venous blood measured by One Touch SureStep Plus glucometer (Lifescan, Milpitas, CA, USA) was higher than 16.7 mmol/L (300 mg/dL) were considered as qualified diabetic models. Six rats were excluded from the study after confirmation of success of diabetic models because of low blood glucose levels. The rest of rats were randomized into two weight-matched groups (10 in each group): diabetic rats with SMF exposure group (SMF group) which were exposed to SMF by gluing one magnetic disk with 230 mT intensity and diabetic rats with sham SMF exposure group (Sham group). 10 normal Wistar rats were used as the Control group.

The current study was performed in adherence to the National Institutes of Health guidelines for the use of experimental animals, and all animal protocols were approved by the Committee for Ethical Use of Experimental Animals of the Yangtzes University.

### 2.2. Surgical Procedure and SMF Apparatus

Two weeks after establishment of diabetes, all rats were anesthetized with administered 50 mg/kg Pentobarbital Sodium. Standardized wounds were created on the backs of 30 rats. These wounds measured 2.0 × 2.0 cm and were produced under sterile conditions by excising skin, subcutaneous tissue, and panniculus carnosus. After achieving hemostasis, the wounds were covered with sterile gauzes. All surgical procedures were performed by the same investigator. For postoperative analgesia, beginning on the day of operation, 0.02 mg/kg fentanyl citrate (Enhua co., Xuzhou, China) was administered subcutaneously, 2 times daily, for 3 days. Rats were housed individually in plastic cages. The distance between cages was 30 cm, to prevent interaction between magnets. The sterile gauzes were replaced once a day. If the wound appeared infection, debrided the infected tissue and applied erythromycin ointment (Mayinglong co., Wuhan, China).

On the day after operation, all diabetic rats in SMF group had permanent NeFeB Magnetic disk (Solectron magnets co., Hangzhou, China) measuring 3.0 × 3.0 cm placed over the wound directly on top of the gauzes (N pole toward the gauzes, The magnetic field strength at the site was 230 ± 5 mT, measured by gaussmeter). The other 10 diabetic rats in sham group had nonmagnetized disk placed.

### 2.3. Healing Parameters

#### 2.3.1. Comparison of the Wound Area Reduction Rates

For surface area measurement, rats were anesthetized with ether inhalation, and then digital camera was used to photograph the wound of rats on postoperative day 7, 14 and 21. Keeping the lens from the target distance for 10 cm when took photos. Then the borders of the skin defect in the pictures were marked, and then the number of pixels within the bordered area was measured to calculate the wound area by using Photoshop CS5 software (Adobe, San Jose, CA, USA). Based on the measured wound area, the wound area reduction rate was calculated by means of the following expression: (former size of the wound-current size of the wound)/(former size of the wound) × 100% [6].

#### 2.3.2. Comparison of Mechanical Strength of Wound Tissue

Postoperative day 21, five rats in each group were randomly selected to be killed by anesthetic overdose and assessed for the dorsal pelt containing the healing scar was removed and cut at a right angle to the long axis of the wound into four 10-mm wide strips. The strips were placed in a buffered Ringer's solution (pH 7.4) and used within 30 minutes of recovering the pelt to assess breaking strength. Biomechanical tests were performed using an electronic universal testing machine (model INSTRON-5840, USA). Two sides of the strips were placed into custom-made mechanical grips. Grip length was selected as 10 mm on both sides and fine-grade sandpapers were placed inside the grips to prevent slipping. Test length of the slips was selected at 30 mm. Tests were performed at a constant speed of 1 mm/minute until breakage at the healing scar was observed. Force was measured with a 260-N load-cell attached to the testing frame.

#### 2.3.3. Comparison of the Mean Time to Wound Closure

The wounds were allowed to heal by secondary intention and the time to complete closure was recorded for the rest five rats in each group.

### 2.4. Statistical Analysis

Statistical analyses were carried out using SPSS (version 14.0, SPSS, IL, USA). Continuous variables were expressed as mean values ± SEM. The values were analyzed by one-way ANOVA and multiple comparisons. In a multiple comparison, difference between two groups was compared using the Student Newman Keuls-*q* test (SNK-*q*). *χ*^2^ test for categorical dates. When *P* was less than 0.05, it was regarded as statistically significant.

## 3. Results

### 3.1. Comparison of Blood Glucose Level

**Figure 1:**
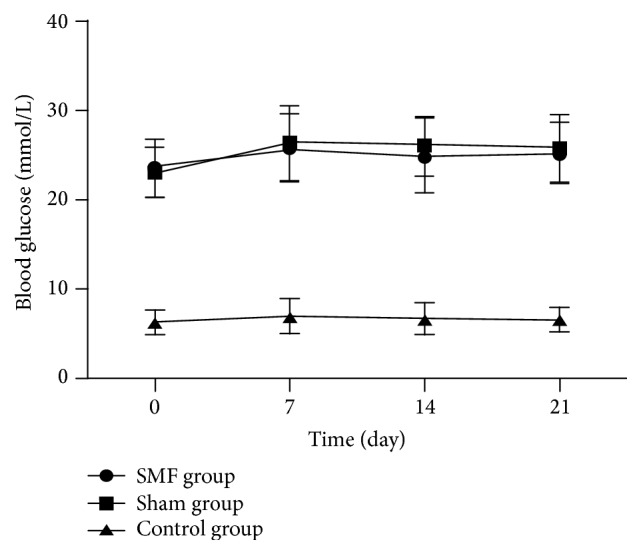
Trends of blood glucose levels in Control, SMF and Sham groups on days 0, 7, 14 and 21 after surgery.

As shown in Figure 1, in the process of the whole experiment, there were no statistical difference between the two diabetic groups (*P* > 0.05).

### 3.2. Comparison of Wound Area Reduction Rate

Table 1 showed that the SMF group and Sham group presented significant visual wound healing delayed compared with the Control group (*P* < 0.01, one-way ANOVA and *q* test). Meanwhile, wound area reduction rate in group SMF at 7th, 14th and 21st days, respectively, was (25.5 ± 4.6)%, (63.4 ± 5.5)%, and (89.7 ± 5.2)%; they were significantly higher than those in sham magnet group (*P* value, resp., was 0.026, 0.005, and 0.001, *q* test).

### 3.3. Comparison of Mechanical Strength of Wound Tissue

**Figure 2:**
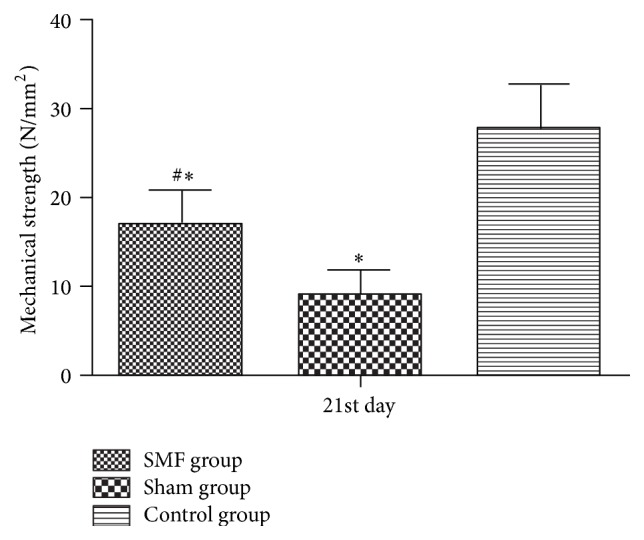
Comparison of mechanical strength in three groups on 21st day, ^#^*P* < 0.05, statistically significant compared to the Sham group; ^*∗*^*P* < 0.01, statistically significant compared to the Control group.

Figure 2 showed that the mean skin wound mechanical strength values for group SMF, sham, and control, respectively, were 16.9 ± 3.8 N/mm^2^, 8.8 ± 2.7 N/mm^2^, and 26.7 ± 5.3 N/mm^2^ (five rats were killed in each group). The stress values for diabetic rats were significantly lower than the control group (*P* < 0.01, one-way ANOVA and *q* test), and use of SMF increased the stress value in diabetic rats (*P* = 0.012, *q* test).

### 3.4. Comparison of the Mean Time to Wound Closure

**Figure 3:**
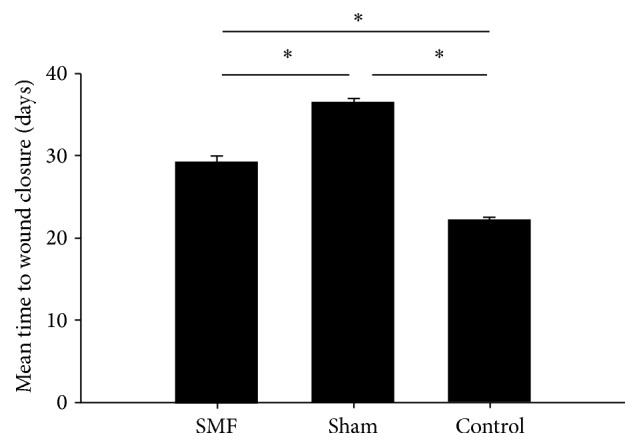
Comparison of mean time to wound closure in three groups,^  *∗*^*P* < 0.05, statistically significant compared to other group.

The wound of partial diabetic rats was healed by second intention due to wound infection: one rat in SMF group, two rats in sham group, zero in control group; there was no significant difference between two diabetic groups (*χ*^2^ = 0.480, *P* = 0.490). As shown in Figure 3, the mean time to wound closure in the group treated with magnets was 29.5 ± 3.8 days compared with 36.5 ± 4.4 days for the sham magnet group and 22.3 ± 2.5 days for the control group (five rats were reserved in each group). Duration of healing time in diabetic rats was significantly greater than normal (*P* < 0.01, one-way ANOVA and *q* test). Application of SMF in diabetic rats significantly (*P* = 0.028, *q* test) reduced the mean time to wound closure.

## 4. Discussion

The present study suggested that exposure to a static magnetic field of 230 mT intensity significantly increased the rate of cutaneous wound healing and reduced the mean time to wound closure (*P* < 0.05). SMF exposure did not present overt impact on serum glucose of diabetic rats throughout the present experiment. It suggested that the capacity of SMF to promote wound healing was not dependent on the changes of serum glucose. These results were similar to Jing et al. previous research result [7]. But the wound healing rate and wound closure in our study were obviously slower than Jing et al. result; this may be due to the difference of intensity of magnetic field, wound dressing, and grouping method: higher magnetic field strength in our study (230 mT versus 180 mT); the wound dressing, respectively, was sterile gauze or hydrogel; we continuously observed wound healing process of each rat; however, only partially rats which executed every once in a while were observed in previous research [7]; the wound of partial diabetic rats was healed by second intention in our study.

In general, the wound healing process can be classified into three different phases: the inflammatory phase, proliferative phase, and remodeling phase. This process is a series of complicated reactions and interactions among cells and mediators, which can be affected by various factors [8]. Diabetes-induced impairment of wound healing is characterized by inhibition of inflammatory response, angiogenesis, fibroplasias, defects in collagen deposition, and differentiation of extracellular matrix. All these have been suggested to contribute to the observed impairment of diabetic wound healing. At present, the conventional treatments for diabetic wounds include platelet-derived products, epidermal growth factor, negative pressure suction, hyperbaric oxygen, and new type medical wound dressings. However, the clinical efficacy of these methods is controversial and some of the treatments (such as platelet derived products, antimicrobial dressings) are of poor cost-effectiveness [9]. As alternative noninvasive method, electromagnetic therapy has been used in the treatment of diabetic wound healing, but it is limited to basic research at present time. One of the findings showed that low-frequency pulsed electromagnetic field (PEMF) accelerated skin wound healing in diabetic rats [10]. But there is a problem with applying PEMF in experimental conditions because they cannot be focused on a specific target tissue. The coils wrapped around the cage that generates the magnetic field affect the animal's entire body. Animals placed in cages have stable positions according to the vector of a pulsed electromagnetic field. To lessen the stress of test animals, exposure times must be limited. Application of SMF is simple, and it achieves a permanent magnetic effect and a permanent vectorial effect. SMF is not related to electric energy, as no heat and electricity harm the tissues. At the same time, the magnetic force applied locally, but not to the whole body or surrounding tissues, has minimal exposure. This makes the SMF a useful tool for the long term. Medical applications of SMF have been generally reported as successful in musculoskeletal disease [11]. Investigations have elucidated the numerous actions of electromagnetic energy on bone including effects on cellular calcium and calcification, collagen and proteoglycans, and angiogenesis. Clinical investigations proved the benefit of electromagnetic therapy in the treatment of delayed unions, difficult fractures, and osteotomies. Animal experiments had shown that moderate field strength of SMF effectively increased the rate of wound healing; the coverage is from 15 mT to 350 mT [12, 13]. Recently, Ekici et al. study showed that high-power static magnetic fields (3900 to 4200 mT) which were placed perpendicular to the wound also increased wound tissue strength in the skin of the experimental model [14].

Mechanical strength is an important measure because it best describes the mechanical property of skin. This parameter of the skin also exhibits a progressive increase in continual tissue repair during the wound healing process [15]. In the present study, we also found that a 230 mT static magnetic treatment of the wound increased the wound breaking strength compared with sham magnet group. It implied that SMF could help to improve the quality of wound healing; similar results had also been reported in previous studies [7, 15].

The mechanism of magnetic field promoting wound healing is not very clear. Synthesizing relevant literature materials [16–18], it may include mainly three aspects: (1) the magnetic field has certain anti-inflammatory effects; (2) magnetic field can promote vascular endothelial cell proliferation and promote the formation of the epidermal neovascularization; (3) magnetic field can promote the formation of skin collagen to promote skin regeneration. In present study, we observed SMF increased wound healing in diabetic rats, but we had not compared it with conventional treatments in curative effect and explored its probable mechanism. These will be the direction of our next research.

In conclusion, according to our findings SMF of 230 mT intensity seems to improve wound healing in diabetic rats. This may provide a noninvasive therapeutic tool for impaired wound healing in diabetic patients.

## Figures and Tables

**Table 1:** Comparison of wound healing rate between three groups ($\overset{-}{x}\pm s$, %).

Group	7th days (*n* = 10)	14th days (*n* = 10)	21st days (*n* = 10)
SMF	25.5 ± 4.6^#,*∗*^	63.4 ± 5.5^#,*∗*^	87.7 ± 4.6^#,*∗*^
Sham	20.3 ± 4.1^*∗*^	52.9 ± 5.2^*∗*^	66.5 ± 7.3^*∗*^

Control	38.6 ± 3.7	86.1 ± 4.7	95.2 ± 1.8

^#^ *P* < 0.05, statistically significant compared to the sham group; ^*∗*^*P* < 0.01, statistically significant compared to the control group.
